# HIV/AIDS Knowledge of Undergraduate Students at a Historically Black College and University

**DOI:** 10.3390/diseases6040098

**Published:** 2018-10-31

**Authors:** Prince Onyekachi Andrew, Azad Bhuiyan, Anthony Mawson, Sarah G. Buxbaum, Jung Hye Sung, Mohammad Shahbazi

**Affiliations:** 1 Public Health Clinical Specialist, Lewisville Medical Pharmacy, Lewisville, TX 75057, USA; princeandrew55@gmail.com; 2 Department of Epidemiology and Biostatistics, Jackson State University, Jackson, MS 39213, USA; anthony.r.mawson@jsums.edu (A.M.); sarah.g.buxbaum@jsums.edu (S.G.B.); jung.h.lee@jsums.edu (J.H.S.); mohammad.shahbazi@jsums.edu (M.S.)

**Keywords:** HIV, AIDS, students, African Americans, HBCU, Mississippi

## Abstract

Objective: This study among 400 undergraduate students enrolled at Jackson State University (JSU) study aimed to assess knowledge about HIV and AIDS among African-American undergraduate students attending a historically black college and university. A cross-sectional survey was conducted. Data were collected using a validated, self-administered, and standardized questionnaire on knowledge regarding risks for HIV and AIDS. Three hundred and eighty-six students (96.5%) had good knowledge about HIV and AIDS, although some participants had misconceptions about the modes of HIV infection transmission. There were no significant gender differences for HIV and AIDS knowledge among the participants (χ^2^ = 3.05; P = 0.08). In general we concluded that JSU undergraduate students had adequate knowledge about HIV transmission modes and AIDS, although some participants had misconceptions about the routes of HIV infection transmission. Hence, this study calls for strengthening HIV and AIDS awareness education among undergraduate students.

## 1. Introduction

HIV continues to be a major public health concern that has claimed more than 34 million lives globally. It is estimated that, in 2016, 1.0 million people died from HIV-related causes globally. In 2016, 36.7 million people were living with HIV, and additional 1.8 million people were infected with the virus [1,2]. In 2015 the US Centers for Disease Control and Prevention (CDC) estimated that 1.2 million people in the US were infected with HIV, and that, globally, one in seven were unaware that they were infected with HIV [3]. The same year, 39,513 people were diagnosed with HIV infection among gay and bisexual men, the population most affected by HIV/AIDS. Gay and bisexual African-American men accounted for the largest proportion of HIV diagnoses in 2015 [3]. Despite representing only 12% of US population, African Americans accounted for 45% (17,670) of HIV diagnoses in 2015. Additionally, in the same year, African Americans had the highest rate of HIV diagnoses compared to other races and ethnicities in the United States [3].

Globally, the sex differences in the incidence of new HIV infections are most pronounced in the younger ages [1]. In 2016, new infections in young women (aged 15–24 years) were 44% higher than in men for the same age group [1]. In the United States, most new HIV infections occur among men who have sex with men (MSM) of all races and ethnicities, followed by African-American heterosexual women [4]. In United States, the CDC estimated that about 22% of all new HIV infections in 2015 were among youth aged 13 to 24 years [4]. Among the youth in the same age group, about four in five of new HIV infections occurred in males, and African Americans accounted for 60% of new infections among youth. Among young gay and bisexual males, African Americans account for over half (54%) of new HIV infections [5]. The CDC also estimates that the majority (87%) of HIV positive young males became infected from contact with MSM, 6% from heterosexual sex, 2% from injection drug use and about 5% from dual exposure to MSM and injection drug use [5]. Among young females, about 86% of HIV infections are associated with heterosexual sex and 13% from injection drug use. New incidence of HIV infections was higher among African American males than in any other group of youth by race/ethnicity and sex [5].

Although sexually transmitted diseases (STDs) affect all ages and races, STDs have taken the greatest toll among young people in the United States [6]. The CDC estimates that although youth ages 15–24 constitute just over one fouth of the sexually-active population, they account for over nine million new sexually transmitted infections that occur in the United States each year [6,7]. Many young people in the US are unaware of their HIV infection status; the CDC estimates that 44% of young people aged 13–24 who were living with HIV do not know their status, which represents the highest rate of undiagnosed HIV in any age group in the United States [8].

Youth often must struggle with their social autonomies, peer pressures and their lack of effective maturity to make positive sexual decisions, which often leads to having negative attitudes and behaviors that contribute to their high vulnerability to HIV infections [9,10]. HIV and AIDS are not simply a public health challenge, but constitute a societal menace with a devastating impact among young people. CDC points out that an average youth feels invincible and has little fear of becoming infected by HIV. In addition, many youths believe that HIV only affects older age groups in the society [6].

Undergraduate university students as a group are constantly exposed to sexual risk behaviors, whether it is during the transition period from high school to university as freshmen, or as returning students in college [11]. Repeated exposures to sexual risk behaviors also make students more vulnerable to HIV infection. Undergraduate students are very mobile group, and if not protected and preserved from the scourge of HIV and AIDS, they can become dispersal agents for the spread of HIV in society [12]. They may also be at higher risk of engaging in risky sexual behavior, especially if they are under the influence of alcohol or drugs, misconception of the knowledge and severity of HIV and AIDS or lack the necessary maturity in handling negative peer pressure [6]. Against this backdrop, it becomes critical that effective HIV and AIDS intervention strategies among undergraduate university students are implemented and effectively promoted and evaluated.

Research has also shown that increased knowledge about HIV and AIDS may not necessarily lead to positive behavior change, yet knowledge about a disease may be an initial step towards behavioral risk change [12,13,14]. In preparing our young people for adult life ahead of them, education remains an excellent tool for transmitting knowledge about HIV and AIDS prevention. A study conducted by Chaves and colleagues found that providing well-planned education about HIV and sexuality increases knowledge, develops better skills, and raises positive attitudes that can reduce risk behaviors among young people [9]. Knowledge about HIV and AIDS can serve as a tool and guide on policy formulation and the necessary intervention in a fight to reduce the prevalence of HIV and other STDs in our schools [12].

Because young people are a valuable resource of society, it is imperative that they are armed with the HIV and AIDS information to protect themselves from falling prey to this infectious disease. In the absence of an effective cure for this disease, assessing the level of knowledge of HIV and AIDS among university undergraduate students will provide vital information on the students’ knowledge and possible misconceptions of the disease. Thus, we assessed knowledge about HIV and AIDS among undergraduate students at a historically black college and university.

## 2. Materials and Methods

### 2.1. Study Area and Design

We conducted a cross-sectional study study to assess knowledge about HIV and AIDS among African American undergraduate students attending a historically black college and university. The survey was conducted from 10 June 2016 to 30 September 2016. Participants were selected through convenience sampling among undergraduate students of Jackson State University (JSU). JSU is in the city of Jackson and has a population of about 9000 undergraduate students. JSU is the fourth largest institution of higher learning in Mississippi and fourth largest of the Historically Black Colleges and Universities (HBCU) in the US [15]. Jackson is also the state capital of Mississippi.

### 2.2. Study Population and Sampling Procedure

The inclusion criteria for the participants included African-American freshman, sophomore, junior or senior undergraduate students of JSU; who were at least 18 years of age; and consented to participate in the research. A minimum sample size of 369 was calculated using the formulas of Michel [16] and Talbot [17]. The sample size was increased to 400 students to allow for non-response. Students were selected through convenience sampling of freshman, sophomore, junior and senior JSU undergraduate students. Perspective participants were informed about the purpose of study and were encouraged to participate in the study after obtaining the permission and approval of their lecturers before their class sessions. Participants were counseled that this study was completely voluntary, that they could refuse to answer any specific question, and that they may withdraw from the study at any time without penalty or prejudice and were given informed consent letters to read and sign prior the distribution the study questionnaires. Questionnaires were distributed to consenting students at the end of their scheduled class session with cooperation and approval of the lecturer in charge. The study participants were required to complete the questionnaires in the classroom. Study participants took an average of ten minutes to complete the questionnaire. The completed questionnaires were retrieved from each participant before leaving the classroom.

### 2.3. Data Collection Instrument

The HIV and AIDS Knowledge Questionnaire used for this study was adapted and modified for this study from a widely used World Health Organization questionnaire [18] and included additional items that were identified from a literature review of related studies. The self-administered study questionnaire was composed of two parts which addressed: (1) the student’s demographic background, and (2) the student’s knowledge regarding HIV/AIDS. We applied Cronbach’s alpha coefficient ranges from 0–1, with values closer to 1.0 indicating higher internal consistency [16,17]. To validate the study questionnaire, we pretested the draft questionnaire with a group of Jackson State University undergraduate students who were not included in the final study. The final questionnaire validation test showed that the Cronbach’s alpha was 0.78 for knowledge.

Knowledge was assessed using items in the questionnaire including: (1) basic knowledge of HIV transmission, (2) predisposing risk behavior and practices for acquiring HIV, (3) symptoms of HIV and AIDS, (4) treatment modalities for HIV and AIDS, (5) methods for preventing infection, and (6) the impact of other STDs on acquisition and spread of HIV infection.

### 2.4. Scoring

Each correct response was given a score of 1, and a wrong or unsure response was scored 0. Total knowledge scores ranged between 0–21. Knowledge scores from 0 to 10 were considered poor knowledge, while scores more than ten were considered as having “good knowledge” regarding HIV and AIDS. 

### 2.5. Data Analysis

Analyses were conducted using SAS^®^ 9.3 statistical software (SAS Institute Inc., 2012). Descriptive statistics gave a comprehensive picture of background variables including age, sex and other dependent and independent variables in the questionnaire. The frequency distribution of both dependent and independent variables was determined. A significance level of α = 0.05 was used for analysis. The Chi-square test was applied for determining an association between knowledge and gender.

### 2.6. Ethical Approvals

Ethical clearance for the study was obtained from the Jackson State University Institutional Review Board. Prior to data collection, all study participants were informed about study and were assured that all data were confidential and will only be analyzed as aggregates. Written informed consent was obtained from each participant prior to enrollment.

## 3. Results

### 3.1. Students’ Profile

Four hundred undergraduate students agreed to participate in the study and completed the questionnaire. The mean age of the 400 respondents was 21.9 years, standard deviation ±5.7 years with ages ranging from 17 to 57 years (Table 1). A total of 141 (35.2%) were male undergraduate students, and 259 (64.8%) were female. All respondents were African American. A total of 88.25% of respondents were Christian and 11.75% indicated no religion.

### 3.2. Knowledge about HIV/AIDS

The data analysis revealed that most respondents (97%) had “good knowledge” about how HIV is transmitted (Table 2). They were aware that HIV is transmitted through sexual intercourse and sharing of injection equipment and that the infection and the disease it causes has become a global pandemic. The respondents were also aware that disease affects the immune system making them susceptible to opportunistic infections and to developing cancers.

More than 97% of the respondents knew that HIV could be transmitted via sharing unsterilized needles and sharps, unprotected sexual intercourse, and through infected semen. More than 90% of the respondents knew that HIV could be transmitted by receiving infected blood, as well as vertical transmission from infected mother to child. Only 71% of respondents thought that consistent use of a condom could prevent HIV transmission during sexual intercourse. However, 87% of the students were aware that HIV infection could be prevented through sexual abstinence, and about 85% of students knew that HIV is a type of virus. On the question of whether HIV and AIDS affect the immune system of infected individuals, about 93% gave an appropriate response; and 88% knew that HIV is already a pandemic disease (12% did not know). On the question whether HIV and AIDS have the same clinical manifestations, about 35% gave an appropriate response. About 74% of the students correctly believed that untreated sexually transmitted diseases increase HIV infection risk, 95% knew that having multiple sex partners can increase the chance of acquiring HIV infection, but only 48% believed that avoiding alcohol and drug abuse reduces the risk of HIV infection.

There were some misconceptions relating to HIV and AIDS, with 55% of respondents believing that HIV could spread through insects or mosquito bites, and about 6% believied that HIV-positive people could be recognized by their facial appearance. Twenty percent or respondents believed that HIV could spread from sharing a bathroom and clothes, and about 22% of the students believed that HIV could spread by sharing public toilet with an infected person. Four percent of the respondents believed that HIV did not affect young people, and 19% believed that there was a cure for AIDS.

The overall mean knowledge score for the 400 respondents in this study was 16.72 ± 2.83, and their knowledge scores ranged from 0 to 21. When the sample was stratified into poor knowledge (scores of 0–10) and good knowledge (scores of 11–21), about 94% of male and 98% of the female students had good knowledge about most items about HIV and AIDS in this study (Table 3). The result from Table 3 also reveals that there was no significant difference between the knowledge of the male and female undergraduate students over HIV and AIDS in this study (χ^2^ =3.05; P = 0.08).

## 4. Discussion

### 4.1. Knowledge about HIV and AIDS

The undergraduate students in this study had a relatively high level of knowledge about HIV/AIDS infection with 96.5% of the respondents having total scores above 10. This finding resembles that observed in a study conducted by Maimaiti and colleagues (2010) on the knowledge, attitude, and practice regarding HIV and AIDS among University Students in Xinjiang, China, which found that 74.5% of their respondents had a similar level of knowledge [19].

Our study showed that more than 97% of the respondents knew that HIV could be transmitted via unprotected sexual intercourse, sharing unsterilized needles and sharps, as well as transmission via infected semen. Lönn and colleagues (2007) conducted a similar study among medical students in the Xinjiang Medical University in 2006, which found that majority of the students knew the common routes of HIV transmission are: unprotected sexual contact, mother-to-child transmission, and sharing unsterilized needles [20]. However, Lönn et al. determined, from in-depth interviews of 20 students, that their students’ knowledge of HIV and AIDS transmission modes was superficial, which underscores the need for more and better structured HIV and AIDS awareness programs for prevention of HIV in young adults [20].

Our study results revealed that that some students lacked knowledge about HIV and AIDS in certain key areas. For example, 12.7% of the students in our study did not recognize sexual abstinence as means of HIV and AIDS prevention, and about 19% of our participants considered AIDS as a curable disease. Other misconceptions about HIV transmission risks detected in our study included the sharing of clothes with HIV and AIDS patients (20%), using a public toilet (21.7%) and insect bites (54.5%). Other studies have detected similar misconceptions about HIV infection transmission routes [19,21,22,23].

While it is encouraging to note that most participants in our study showed relatively high levels of knowledge about HIV and AIDS infection, it is disturbing that 4.2% of respondents still believed that HIV does not affect young people, and that 5.7% believed that they could identify an individual with HIV infection by looking at the person’s facial expression. A similar study done by Fennie and colleagues [11] indicated that many adolescents tend not to perceive themselves to be at risk for HIV or other sexual transmitted disease. Fennie and colleagues also found that some adolescents with good knowledge of HIV and AIDS risk do not see the need for safe sex as serious, and they often downplay the risk of HIV infection [11]. In our study, some students were unaware that individuals having other sexual transmitted diseases have a higher risk of acquiring HIV compared to individuals without STDs. This study finding is also consistent with similar findings noted in other studies [19,21,22,23]. Importantly, we noted that there was no significant difference in HIV and AIDS knowledge between male and female students in our study.

Our research and that of others has shown that increased knowledge about HIV and AIDS may not predict behavioral change in all undergraduate students [12,13,14,24], however knowledge about the disease is a prerequisite for the behavioral changes that will protect most students from HIV infection [12,25].

### 4.2. Strength and Limitations of the Study

This study was conducted among undergraduate students from a single college; the findings would have been more generalizable if the students’ knowledge about HIV and AIDS status was evaluated at more than one historically black college and university. The study was also limited by the study design, which made it difficult to differentiate cause and effect from simple association. However, the strength of this study was the nearly 100 percent response rate of from study participants, allowing reliable estimation of the level knowledge of HIV and AIDS knowledge among the University students.

## 5. Conclusions

This study found that a sample of 386 undergraduate students from a historically black College (representing 96.5% of eligible students) possessed high levels of knowledge about risks of HIV and AIDS. Student misconceptions about HIV/AIDS noted in the study included the beliefs that sharing of clothes with HIV and AIDS patients (20%), using a public toilet (21.7%) and insect bites (54.5%) would transmit HIV. These are rare but important misconceptions underscore the need for continuing education interventions to keep young adults informed about HIV and AIDS. This study also points out the need for additional studies to evaluate undergraduate students’ HIV infection risk behaviors and attitudes towards people living with HIV and AIDS. It also points to the imperative for colleges in the United States and other countries to offer health education programs related to HIV and AIDS transmission and prevention for their students. This can help to improve and correct some of the important misconceptions regarding HIV and AIDS.

## Figures and Tables

**Table 1:** Demographic characteristics of the 400 undergraduate students enrolled in the study.

Characteristics	*n* (%) or Mean ± S.D.
Age	21.9 ± 5.7
Gender:	
Male	141 (35.2)
Female	259 (64.8)
Religion:	
Christian	353 (88.25)
Non-Christian	47 (12.75)

*n* = Number of students in each group; S.D. = Standard Deviation; % = Percentage.

**Table 2:** Knowledge about HIV and AIDS among 400 undergraduate students enrolled in the study.

Variables	Appropriate Responses	*n* (%)
HIV is a type of virus	True	339 (84.8)
AIDS is a curable disease	False	324 (81)
HIV/AIDS affects the immune system	True	373 (93.3)
HIV and AIDS have the same clinical manifestations	False	141 (35.3)
Opportunistic infections are common among AIDS patients	True	158 (39.5)
HIV is already a pandemic disease	True	350 (87.5)
People can get HIV from:		
Sexual intercourse without a condom	True	389 (97.3)
Infected mother-to-child transmission	True	377 (94.3)
Receiving infected blood	True	369 (92.3)
Sharing infected needles and sharps, e.g., razor blades, nail cutters, lancet, syringes	True	389 (97.3)
Through infected semen	True	389 (97.2)
HIV infection can be prevented through:		
Consistent use of condoms can prevent HIV Infection	True	285 (71.3)
Sexual abstinence	True	349 (87.3)
HIV misconceptions:		
HIV is transmitted through insect bites	False	182 (45.5)
HIV is transmitted through sharing clothes	False	320 (80)
HIV is transmitted through using public toilet	False	313 (78.3)
Diagnose HIV by looking at facial expression	False	377 (94.3)
HIV does not affect young people	False	383 (95.8)
HIV infection risk:		
Multiple sex partners increase HIV infection risk	True	380 (95)
Untreated STD increases HIV infection risk	True	299 (74.8)
Avoiding alcohol and drug abuse reduce HIV risk	True	193 (48.3)

STD = Sexual transmitted disease; HIV = Human immunodeficiency virus; AIDS = acquired immune deficiency syndrome; *n* = Number of students; % = Percentage.

**Table 3:** Differences in distribution of knowledge about HIV and AIDS for all respondents by sex.

Variables	Knowledge			
	Good Knowledge *n* (%)	Poor Knowledge *n* (%)	χ^2^	P
*Sex*				
Male	133 (94.3)	8 (5.7)	3.05	0.08
Female	253 (97.7)	6 (2.3)		

*n* = Number of students in each group; χ^2^ = Chi-square; % = Percentage.

## References

[1] (2017). UNAIDS Data.

[2] (2017). HIV Fact Sheet.

[3] (2017). CDC Fact: HIV in the United States: At a glance.

[4] (2017). CDC Fact: Today’s HIV/AIDS Epidemic.

[5] (2012). CDC Vital Signs—HIV among Youth in the US.

[6] (2015). CDC Vital Signs—HIV among Youth.

[7] (2015). Sexually Transmitted Diseases.

[8] (2017). HIV among Youth.

[9] Chaves C.B., Bentoa M.T., Ferreira M.C., Duarte J.C. (2013). Knowledge about HIV/AIDS: The influence of lifestyles and self-regulation in adolescents. Eur. J. Couns. Psychol..

[10] Orgiles M., Carratala E., Carballo J.L., Piqueras J.A., Espada J.P. (2013). Factors associated with sex under the influence of alcohol among adolescents with divorced parents. J. Child Adolesc. Subst. Abuse.

[11] Fennie T., Laas A. (2014). HIV/AIDS-related knowledge, attitudes and risky sexual behavior among a sample of South African university students. Gender Behav..

[12] Bigala P., Adebowale S.A., Oladipo S.E. (2014). Influence of HIV testing on knowledge of HIV/AIDS prevention practices and transmission among undergraduate youths in North-West University, Mafikeng. Gender Behav..

[13] Oladipo S.E., Amoateng A.Y., Kalule-Sabiti I. (2014). Psycho-social experiences and coping among caregivers of people living with HIV/AIDS in the North-West province of South Africa. S. Afr. J. Psychol..

[14] Omoyeni S.T., Akinyemi A.I., Fatusi A. (2014). Adolescents and HIV-related behaviour in Nigeria: Does knowledge of HIV/AIDS promote protective sexual behaviour among sexually active adolescents?. Afr. Popul. Stud. Spec. Issue Nigeria.

[15] Historically Black Colleges and Universities (2015). Jackson State Becomes the 4th Largest HBCU by Enrollment.

[16] Mishel M.H. (1998). Methodological studies: Instrument development. Advance Design in Nursing Research.

[17] Talbot L.A. (1995). Principles and Practice of Nursing Research.

[18] World Health Organization (1988). Interview Schedule on Knowledge, Attitude, Beliefs and Practices on AIDS/KABP Survey.

[19] Maimaiti M., Shamsuddin K., Abdurahim A., Tohti N., Memet R. (2010). Knowledge, attitude and practice regarding HIV/AIDS among university students in Xinjiang. Glob. J. Health Sci..

[20] Lönn E., Sahlholm K., Maimaiti R., Abdukarim K., Andersson R. (2007). A traditional society in change encounters HIV/AIDS: Knowledge, attitudes, and risk behavior among students in northwestern China. AIDS Patient Care STDS.

[21] Al-Rabeei N.A., Dallak A.M., Al-Awadi F.G. (2012). Knowledge, attitude and beliefs towards HIV/AIDS among students of health institutes in Sana’a city. Eastern Mediterranean Health J..

[22] Shiferaw Y., Alemu A., Girma A., Getahun A., Kassa A., Gashaw A., Alemu A., Teklu T., Gelaw B. (2011). Assessment of knowledge, attitude and risk behaviors towards HIV/AIDS and other sexual transmitted infection among preparatory students of Gondar town, North West Ethiopia. BMC Res. Notes.

[23] Tavoosi A., Zaferani A., Enzevaei A., Tajik P., Ahmadinezhad Z. (2004). Knowledge and attitude towards HIV/AIDS among Iranian students. BMC Public Health.

[24] Onah H.E., Mbah A.U., Chukwuka J.C., Ikeme A.C. (2004). HIV/AIDS awareness and sexual practices among undergraduates in Enugu, Nigeria. Nigerian Postgrad. Med. J..

[25] Fawole I.O., Asuzu M.C., Oduntan S.O., Brieger W.R. (1999). A school based AIDS education programme for secondary school students in Nigeria: A review of effectiveness. Health Educ. Res. Theory Prac..

